# Common pediatric football (American) related extremity injuries: a pictorial essay

**DOI:** 10.1007/s10140-025-02436-0

**Published:** 2026-02-04

**Authors:** Kalpana Manral, Rachelle Durand, Nirav Pandya, Aaron Kornblith, Kayla Cort, Jesse Courtier, Victoria A. Young

**Affiliations:** 1 Department of Radiology and Biomedical Imaging, University of California San Francisco, Benioff Children’s Hospital, San Francisco, CA USA; 2 Department of Orthopedic Surgery, University of California San Francisco, San Francisco, CA USA; 3 Department of Emergency Medicine, University of California San Francisco, Benioff Children’s Hospital, San Francisco, CA USA

**Keywords:** Pediatric Football injuries; American Football; Extremity injuries, Football

## Introduction

According to a CDC report, between 2010 and 2016, an estimated 2.7 million emergency department visits per year were attributed to sports-related injuries among individuals aged 5 to 24 years. (3). American football (14.1%) was the most frequent sport accountable, followed by basketball (12.5%), pedal cycling (9.9%), soccer (7.1%), and ice or roller skating or skateboarding (6.9%) [1]. Sports injuries also have a significant financial impact on the health system with an estimated costs per year of sports-related pediatric emergency room visits of up to $930 million [2, 3].

A comparative study by Lykissas et al. 3 reviewing pediatric sports related injuries in 2000, 2005, and 2010 suggested that pediatric sports-related injuries decreased in most sports but increased in football by 23%. Another study by Radelet et al. 4 concluded that youth football participants are at much higher risk of encountering injuries than those participating in baseball, basketball, or soccer. [3]

Sports-related injuries have recently increased, particularly in the pediatric age. Despite the availability of protective gear, better techniques, and increased medical care on and off the field, sport-related injuries continue to cause long-lasting morbidity in the young population. Early exposure to sport-specific training can have broad physical, social, and psychological effects on young athletes. It is linked to higher risks of overuse and serious injuries, along with emotional exhaustion and unmet psychological needs. Although many consensus statements caution against early specialization, the exact relationship between this practice and its negative outcomes remains unclear [4].

The purpose of this pictorial essay is to review the commonly encountered injury types from American football seen in pediatric patients and discuss management approaches. Specifically we will focus on non-neurologic injuries as outlined in Table 1.

**Table 1:** Injuries based on anatomical compartments:

Anatomical Location	Injury Pattern
Upper Extremity	Muscle strain Muscle hematoma/bruise Shoulder dislocation Fractures Glenolabral tear Rotator cuff tear Neuritis secondary to overuse injury
Chest	Pectoralis Major muscle tear
Abdomen	Solid organ injuries
Lower Extremity	ACL tear/strain Meniscal tear Hamstring muscle avulsion/tear Fractures/dislocation Osteochondral defect Deltoid ligament/distal syndesmotic injury Morel lavallee lesion

### Upper extremity

A retrospective review of football-related injuries treated at a level 1 pediatric referral hospital emergency department and surrounding urgent care clinics between January 2010 and January 2014 indicated that fractures were the most common type of injury across all age groups. The fracture rate, which had risen steadily to 47.6% among those aged 12–14 years, dropped significantly to 26.2% in the 15–18-year age group. Conversely, soft tissue injuries showed a marked increase in the 15–18-year group**.** [3]

### Shoulder

The glenohumeral joint is a ball-and-socket joint, one of the most mobile joints in the human body, and a commonly encountered injury site. The shoulder is the most commonly dislocated large joint in adolescent athletes, with a spectrum of glenohumeral dysfunction ranging from frank acute dislocation to subacute multidirectional instability [5]. Injuries involving the glenohumeral joint include shoulder dislocation, fractures, labral tears, and rotator cuff tears.

Common types of labral tears include:

- Superior labral anteroposterior tear (SLAP)
- Antero-inferior labral tear/soft tissue Bankart lesion
- Anterior labro-ligamentous periosteal sleeve avulsion (ALPSA)
- Gleno-labral articular disruption
- Perthes lesion

SLAP tear involves the superior labrum and is divided into four subtypes based on Synder classification.

**Fig. 1:**
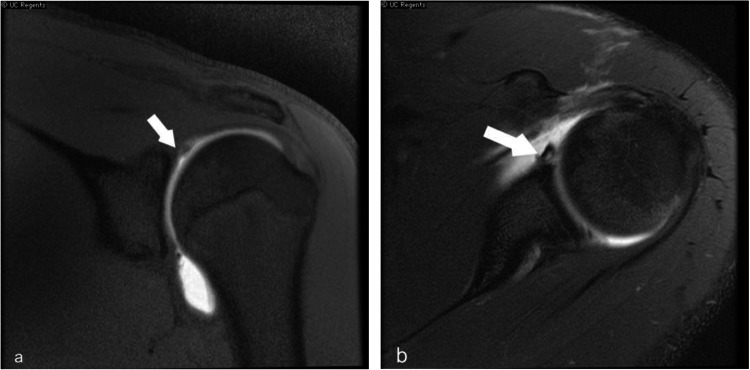
Shoulder MR arthrogram in a 17 year old demonstrates superior labral anterior posterior tear (SLAP). (a) Coronal T2 fat sat image shows superior glenoid labrum tear (white arrow). (b) T2 axial fat sat image from the same patient shows hyperintense tear along the anterior labrum with surrounding joint effusion (white arrow)

**Fig. 2:**
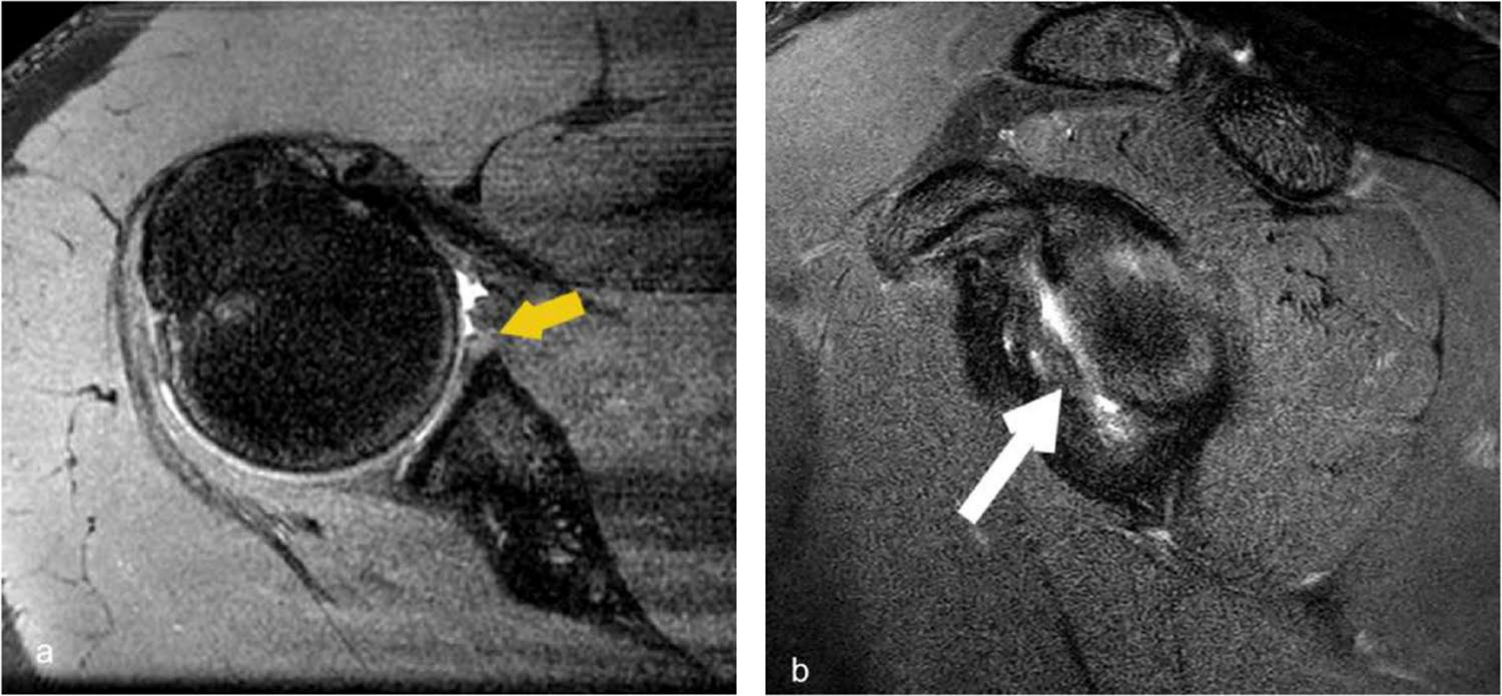
Shoulder MRI demonstrates anteroinferior labral tear/soft tissue Bankart lesion. (**a**) Axial T2 fat sat image shows indistinct anterior labrum with intact glenoid cortex. (yellow arrow) (**b**) Sagittal T2 fat sat image depicts hyperintense tear along the anterior inferior glenoid labrum (white arrow)

**Fig. 3:**
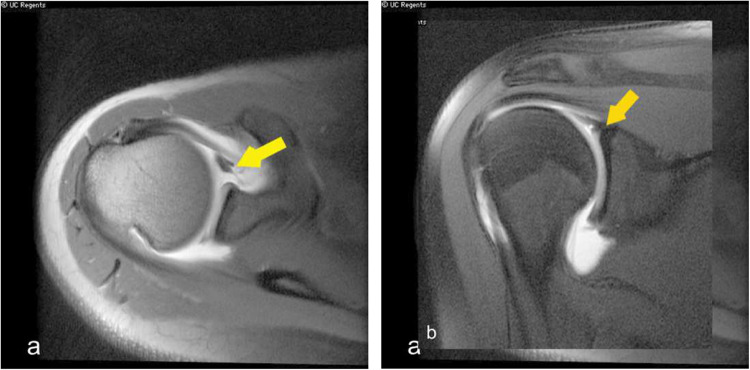
Shoulder MR arthrogram images (**a**,**b**) demonstrate ALPSA, where hypointense labrum and cartilage with stripped periosteum migrates without detaching from the underlying glenoid bone (yellow arrows). Contrast within the shoulder joint space outlines the tear and its extent

**Fig. 4:**
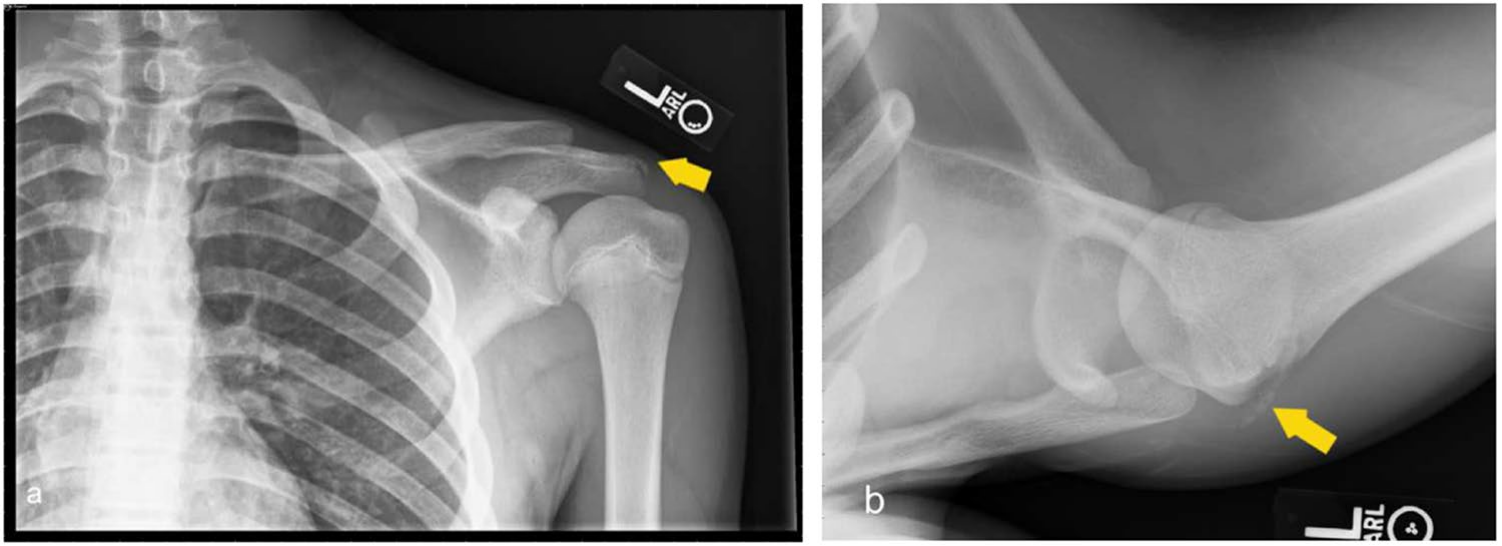
Shoulder radiographs depict periosteal reaction along the acromion suggesting healing fracture of the acromion. Glenohumeral alignment is maintained

**Fig. 5:**
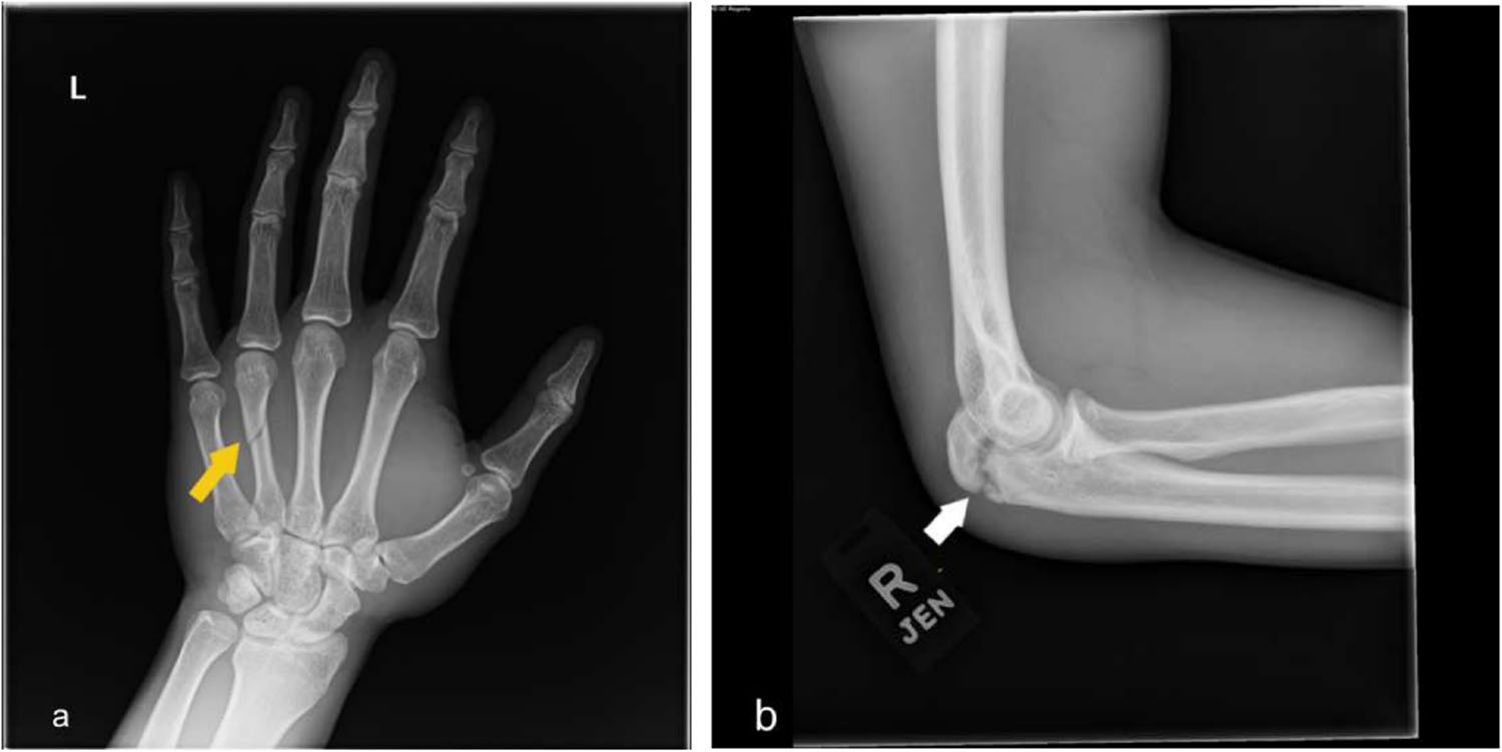
(**a**) Frontal hand radiographs demonstrate spiral fracture of the fourth metacarpal shaft without significant displacement. (yellow arrow) (**b**) Lateral elbow radiograph shows nondisplaced olecranon fracture with maintained elbow joint alignment (white arrow)

**Fig. 6:**
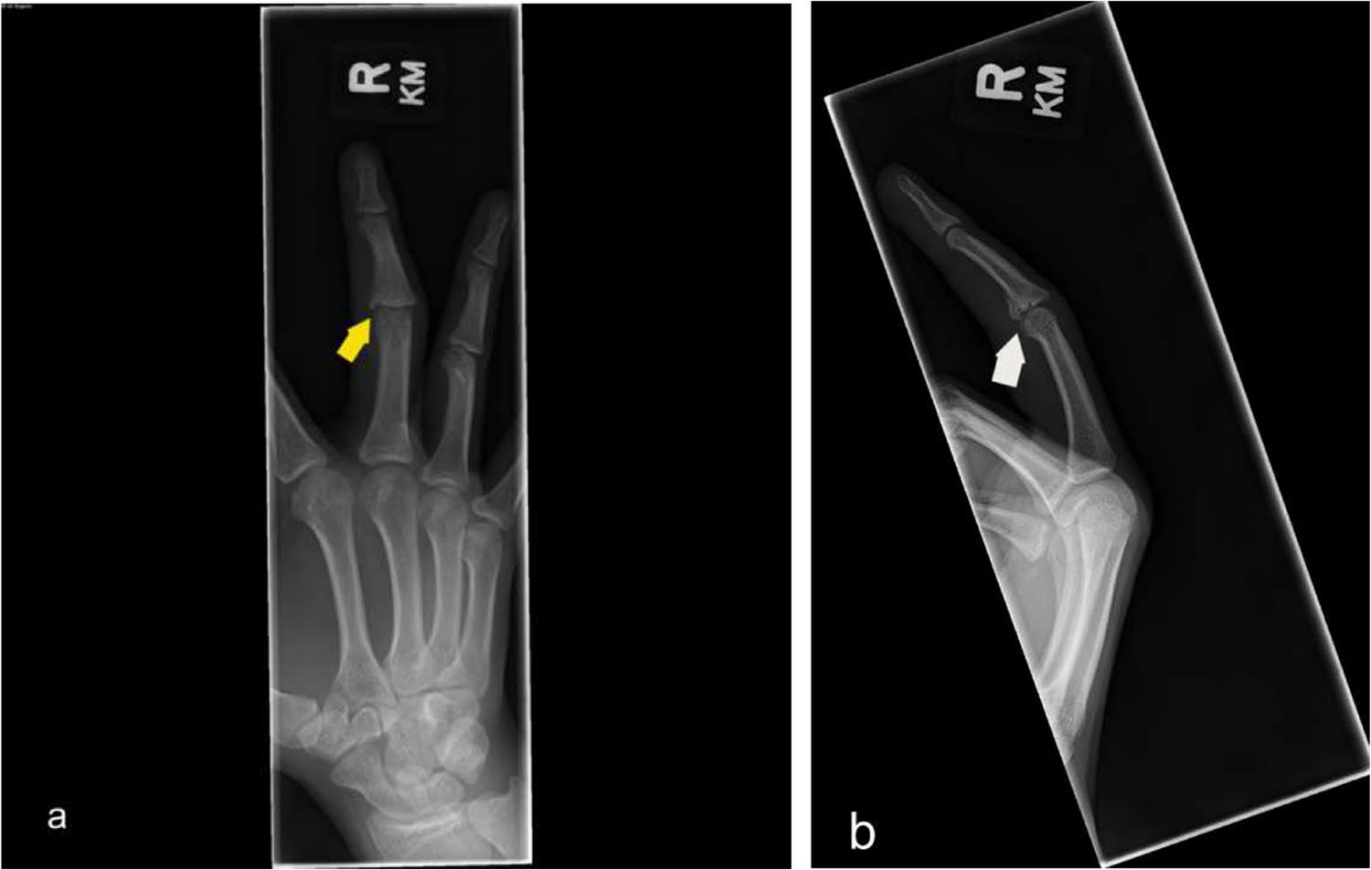
Lateral and oblique finger radiographs show volar plate avulsion fracture with mild displacement of the fracture fragment. Soft tissue swelling about the proximal interphalangeal joint. (white and yellow arrows respectively)

**Fig. 7:**
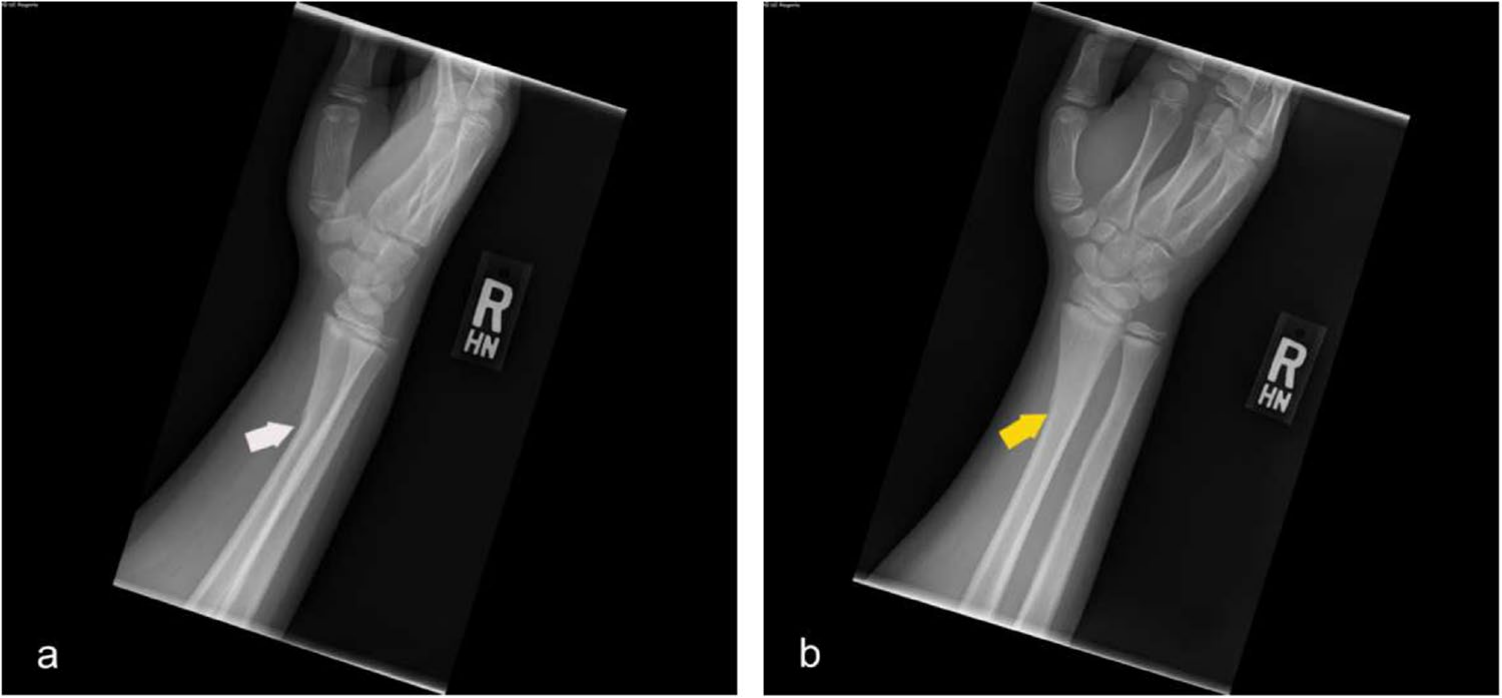
Lateral and oblique wrist radiographs demonstrate cortical buckle along the volar aspect of the distal radius without discrete cortical breach consistent with greenstick fracture. (white and yellow arrows respectively)

**Fig. 8:**
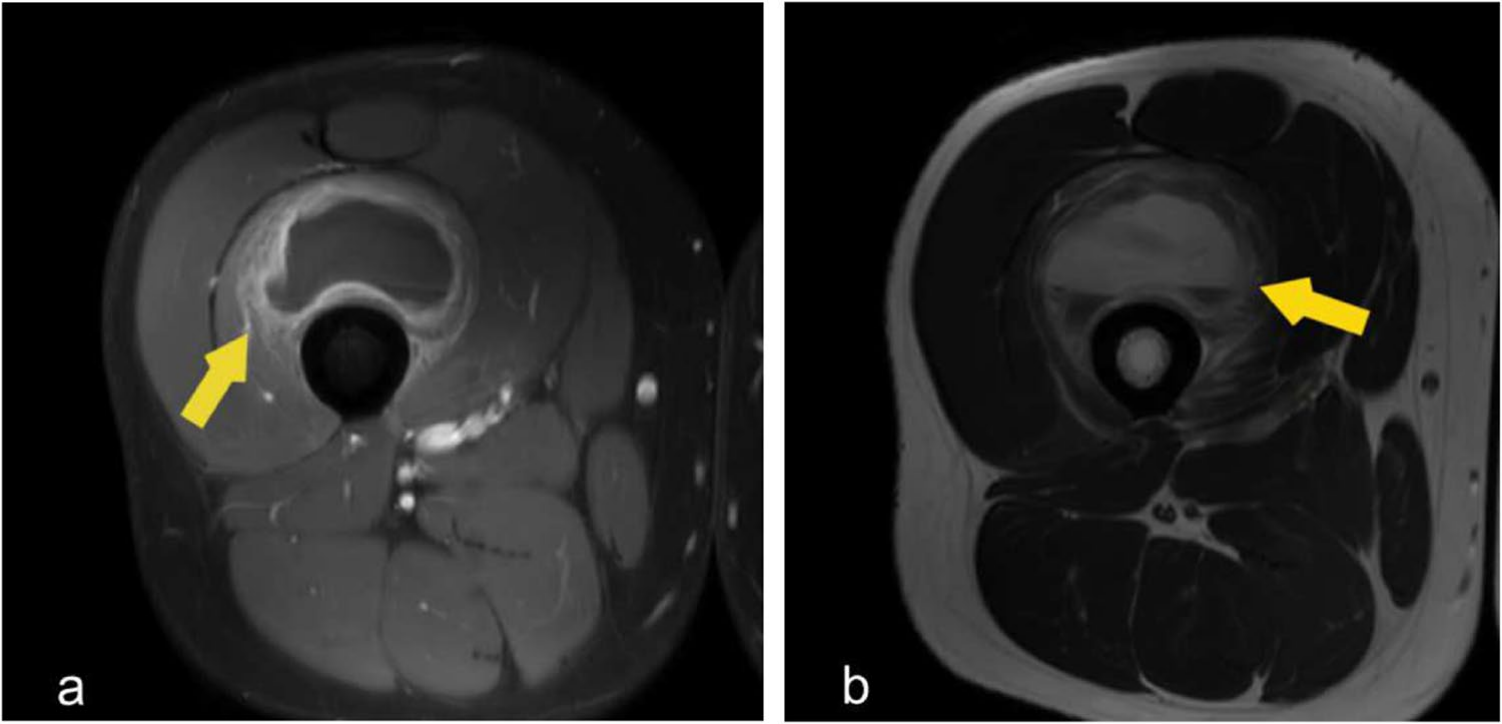
Axial MRI demonstrates muscle hematoma. (**a**) Axial T2 fat sat contrast enhanced MRI knee demonstrates peripherally enhancing collection centered at the vastus intermedius muscle.(Yellow arrow) (**b**) Axial T2 MRI knee in the same patient shows hyperintense collection with fluid fluid level, indicates the hematocrit effect—typically seen in the subacute phase (days–weeks) but can occur earlier or later, especially with rebleeding or in large, relatively static collections.(yellow arrow)

**Fig. 9:**
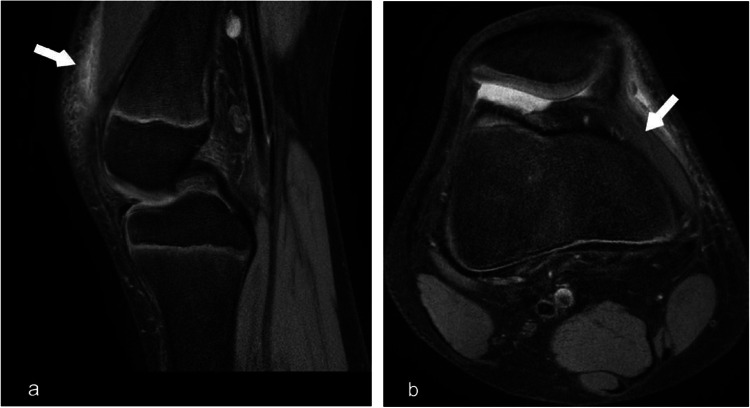
MRI knee demonstrates partial muscle tear/strain. (**a**) Sagittal T2 fat sat MRI knee shows hyperintensity along the vastus medialis muscle (white arrow). (**b**) Axial T2 fat sat MRI knee in the patient again demonstrates hyperintensity along the vastus medialis.(white arrow)

**Fig. 10:**
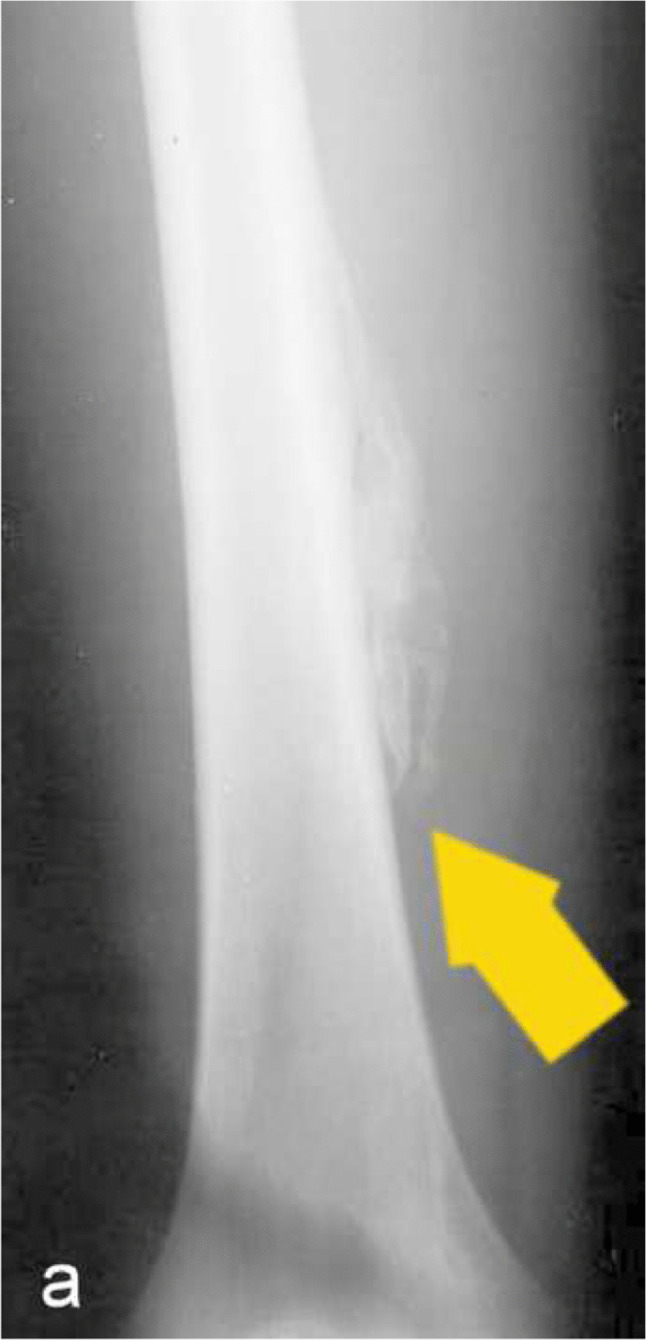
Frontal distal femur radiograph in a 15 year old with a history of football injury one month ago demonstrates increased ossification in the soft tissues along the distal femoral metaphysis consistent with myositis ossificans. (yellow arrow)

**Fig. 11:**
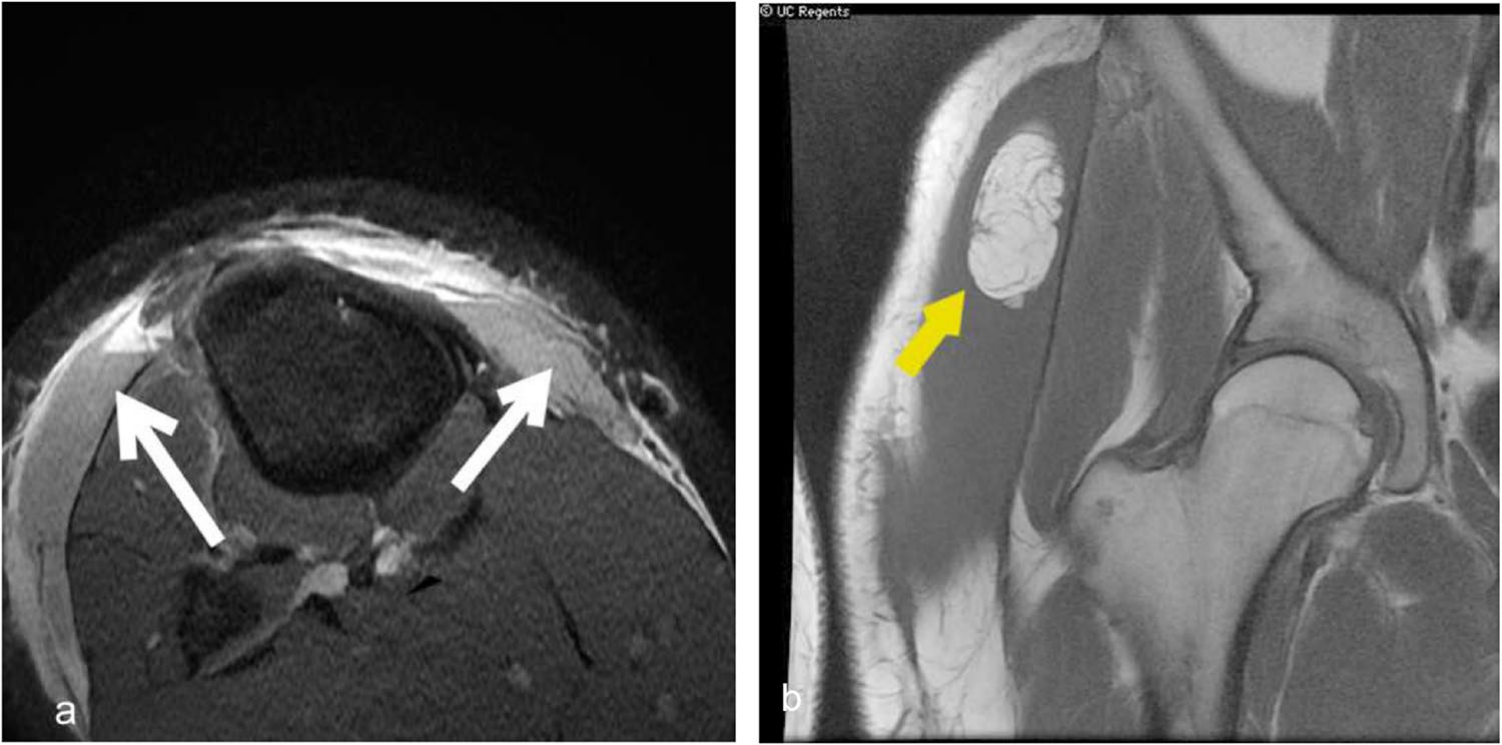
Morel Lavallee Lesion. (**a**) MRI knee demonstrates T2 hyperintense collection along the subfascial plane with fluid–fluid level. (yellow arrow) (**b**) MRI hip shows a large T2 hyperintense collection abutting the muscle plane with internal hypointense focus suggesting internal fat globule.(white arrow)

**Fig. 12:**
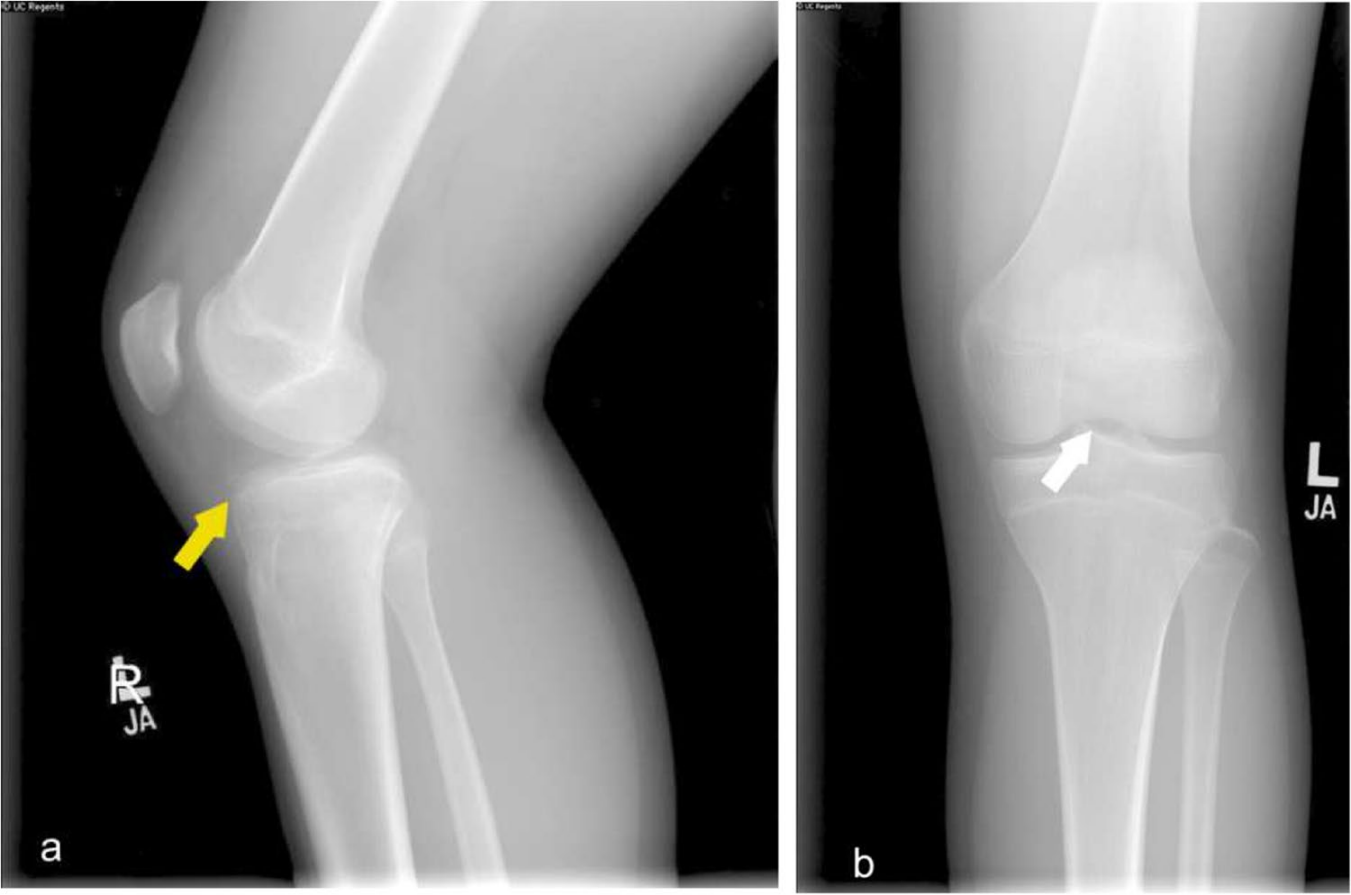
Frontal and lateral knee radiographs depict small avulsed osseous fragments near the expected location of anterior cruciate ligament (ACL) suggesting ACL avulsion injury. Mild soft tissue swelling about the knee

**Fig. 13:**
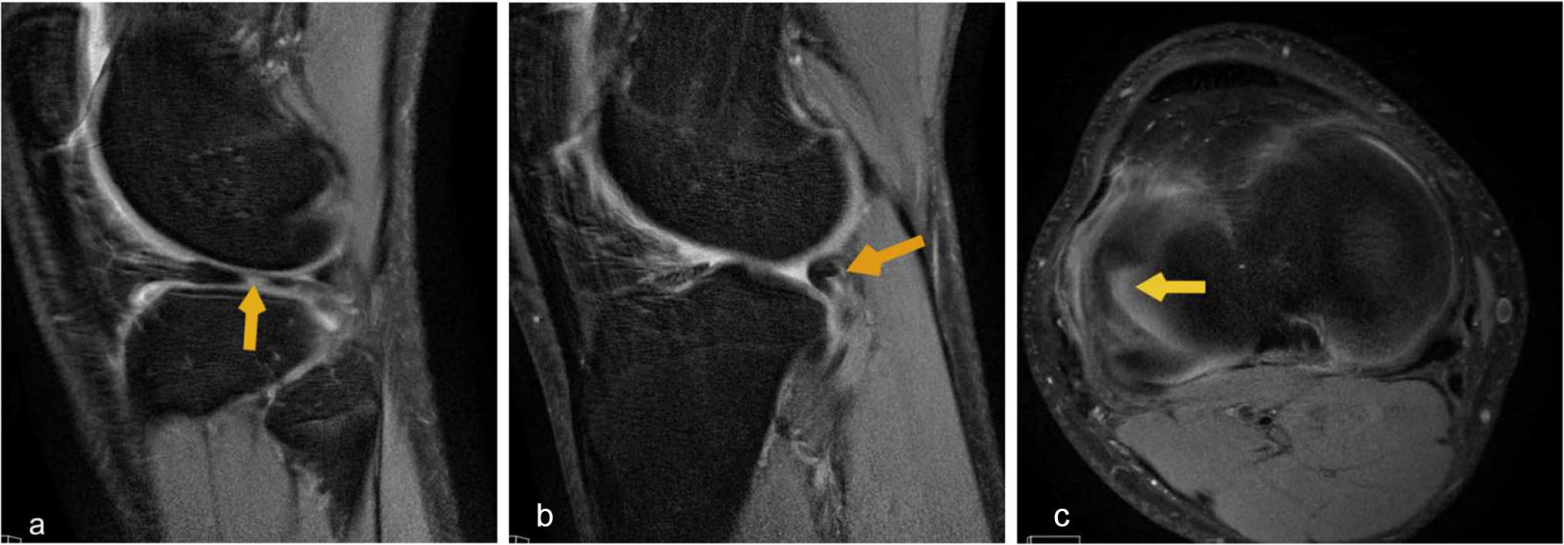
(**a**, **b**) Sagittal T2 knee MRI knee images demonstrate medial meniscus tear with flipped meniscal body posteriorly (yellow solid arrow). (**c**) Axial T2 knee MRI at the level of meniscus shows tear along the medial meniscus (yellow solid arrow)

**Fig. 14:**
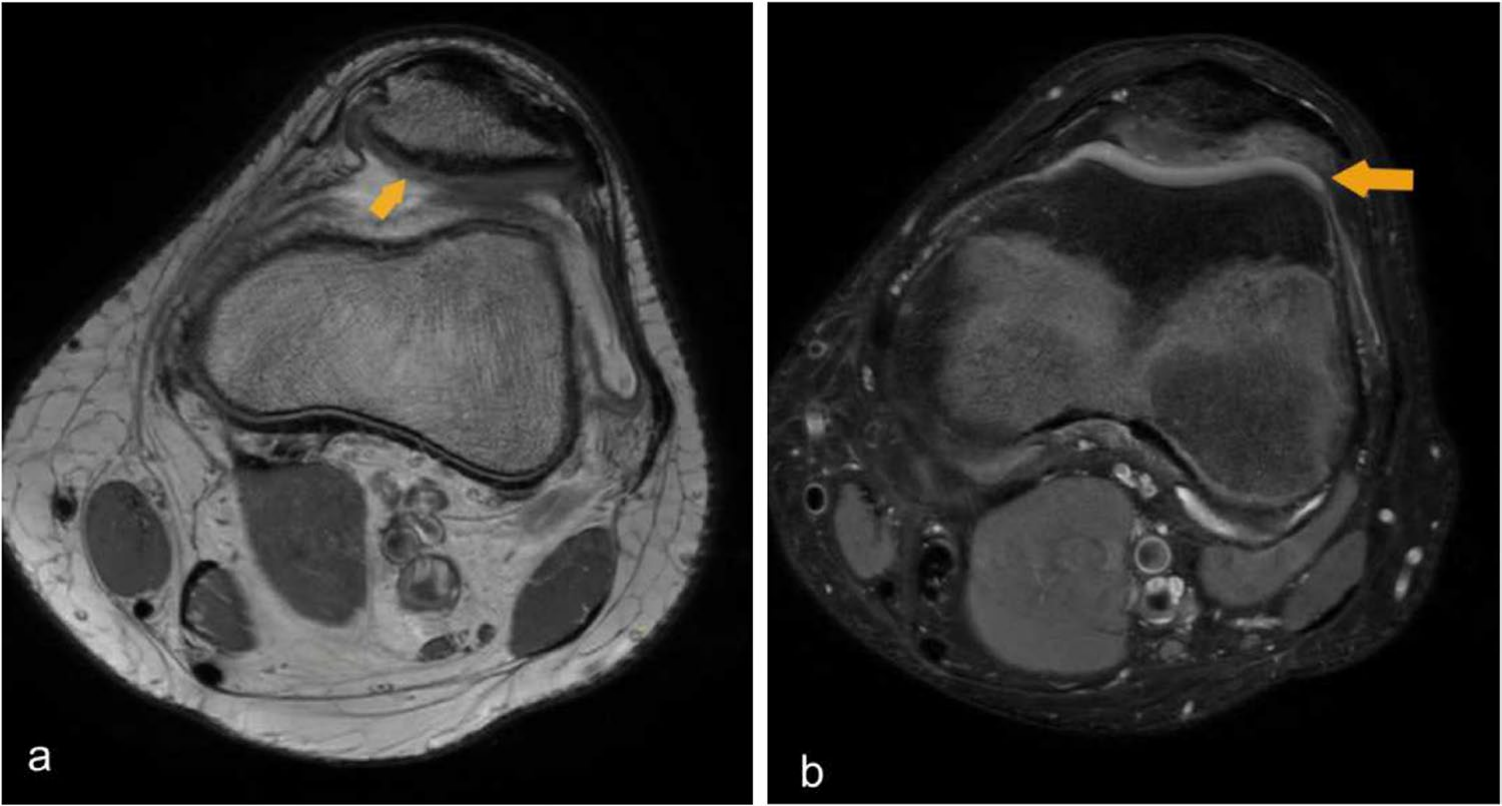
(**a**) Axial T1 MRI knee demonstrates patellar cartilage injury along the medial facet evident as thinned out cartilage (yellow arrow). Axial T2 fat sat MRI knee in the same patient shows increased T2 signal intensity along the inferior lateral patellar cartilage (yellow arrow)

**Fig.15:**
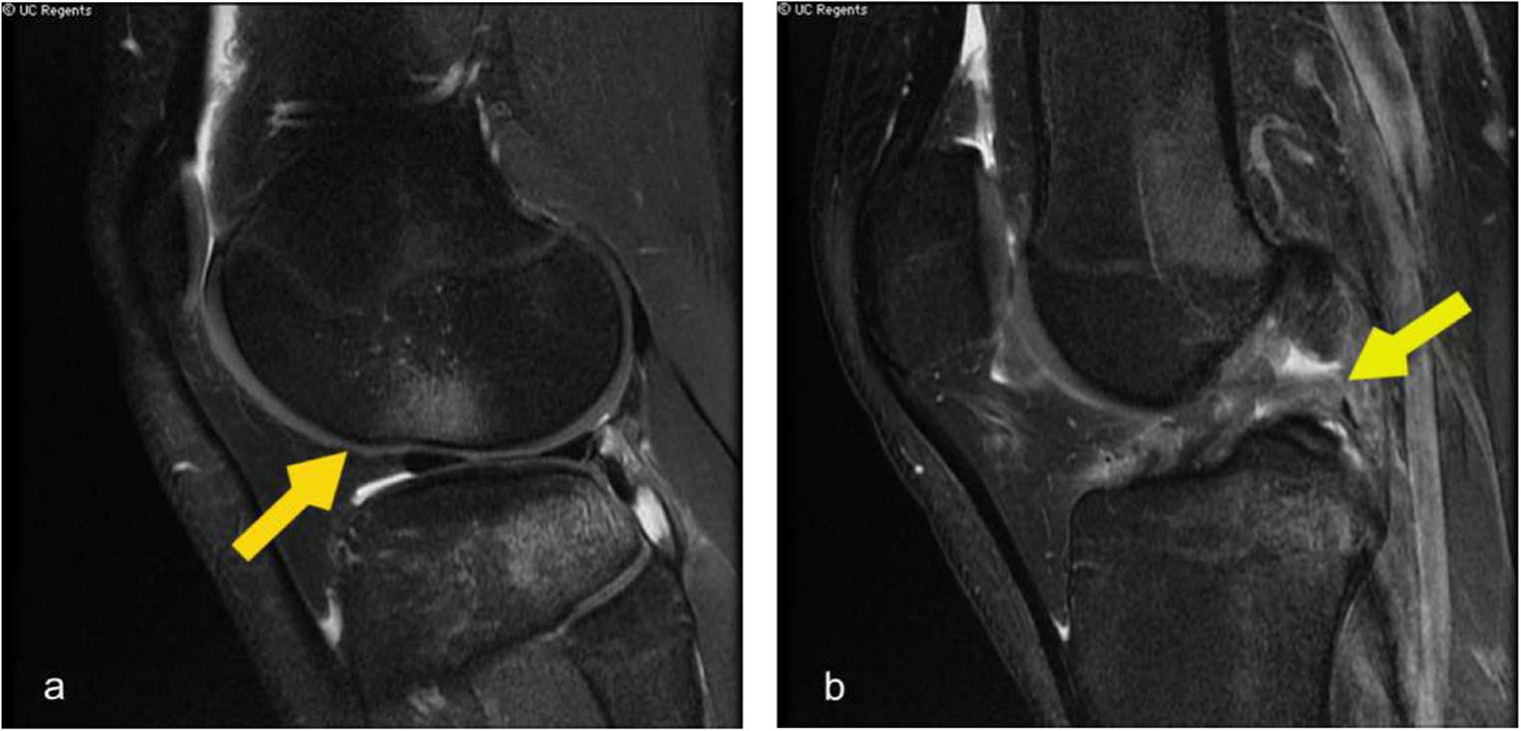
(**a**) Sagittal T2 fat sat MRI knee demonstrates complete tear of the anterior cruciate ligament along the mid substance. (**b**) Parasagittal T2 fat sat plane in the same patient shows bone contusion along the lateral femoral condyle and posterior lateral tibial plateau with associated joint effusion.(yellow arrow)

**Fig. 16:**
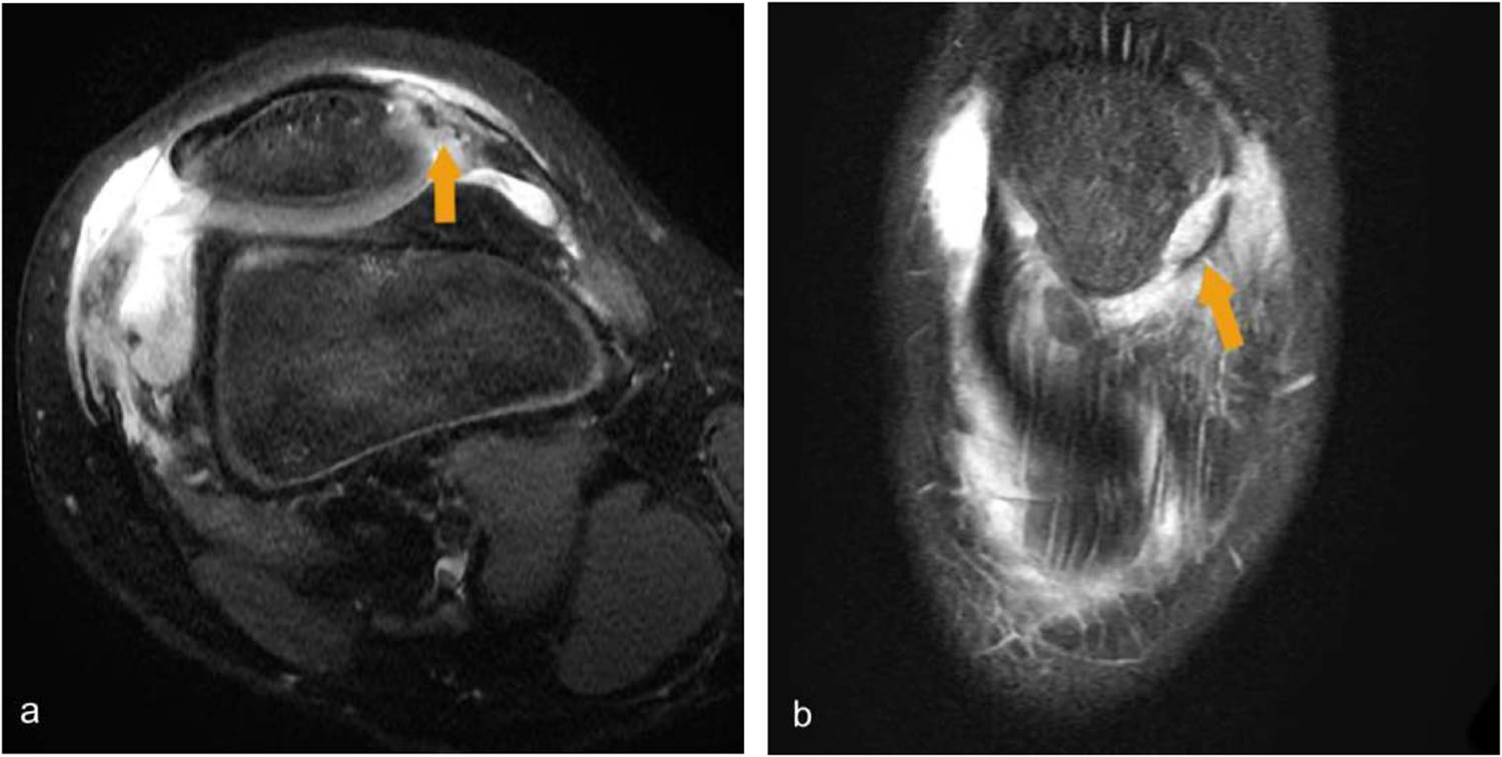
(**a**) T2 fat sat MR knee demonstrates complete tear of the lateral patellar retinaculum with knee joint effusion (**b**) Coronal MRI knee in the same patient shows avulsed lateral patellar facet with lateral patellar retinacular tear. (yellow arrows)

**Fig. 17:**
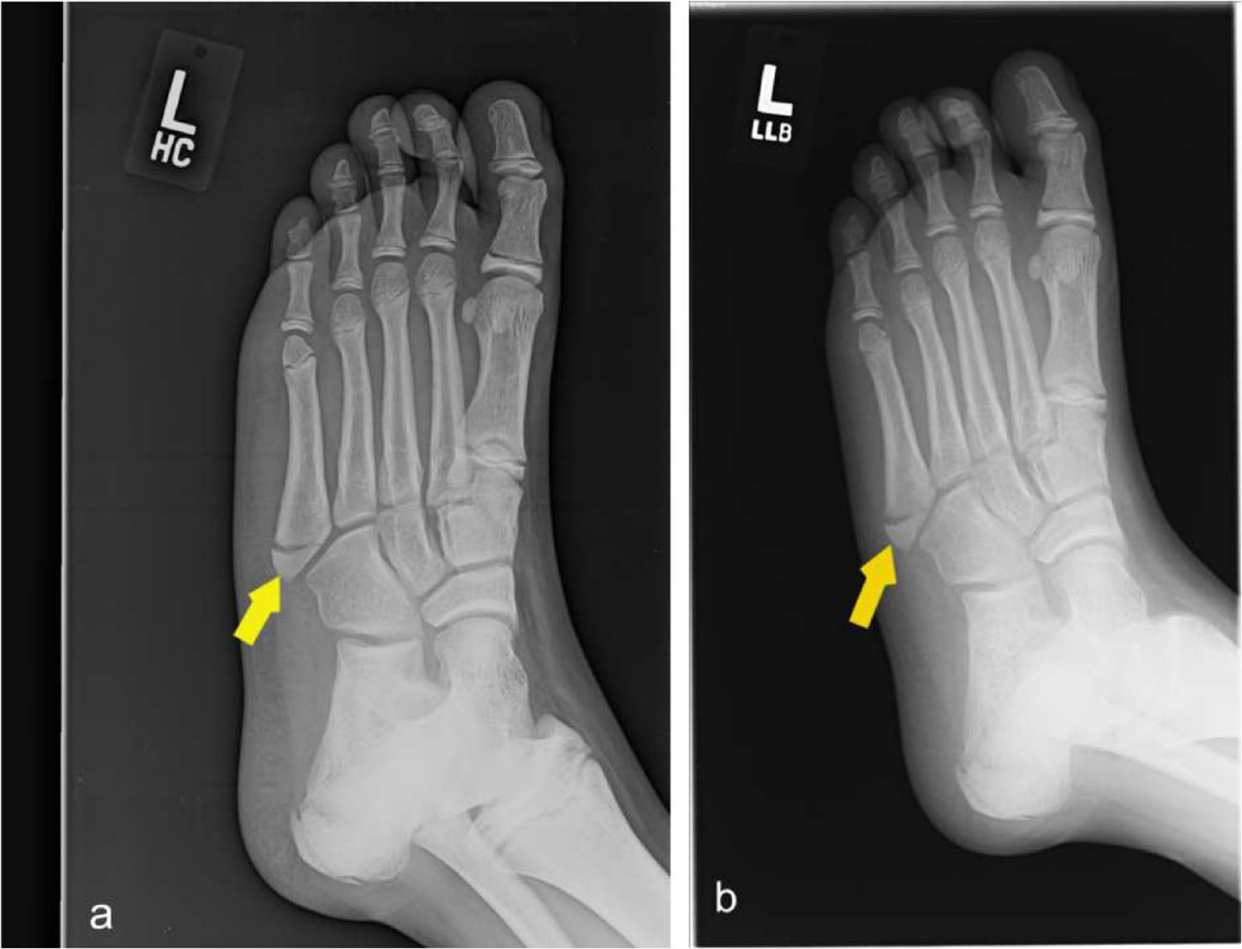
Frontal and oblique radiographs demonstrate minimally displaced fracture of the 5th metatarsal base. (yellow arrow)

**Fig. 18:**
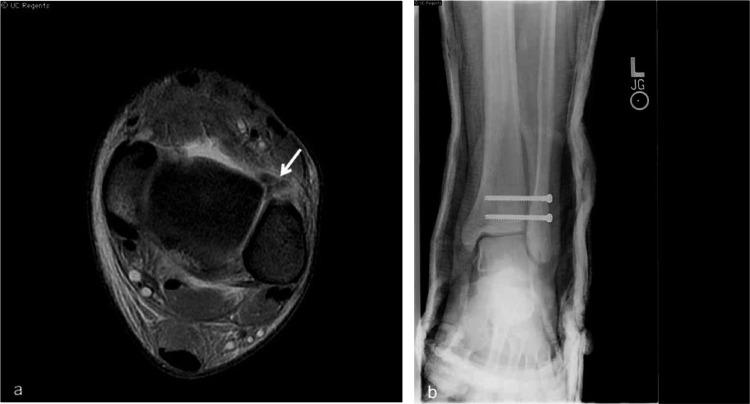
(**a**) Axial T2 fat sat and axial T1 ankle MRI demonstrate complete tear of the anterior talofibular ligament with surrounding fat stranding (white arrow). (**b**) Postoperative radiograph in the same patient demonstrates two screws transfixing the distal tibiofibular joint

Type I: Fraying of the superior labrum without a distinct tear.

Type II: Detachment of the superior labrum and biceps tendon at the chondral-labral junction. Most commonly associated with Bankart lesions in pediatric patients. (Fig. 1).

Type III: Bucket handle tear of the superior labrum without extension into the biceps tendon.

Type IV: Bucket handle tear of the superior labrum with extension into the biceps tendon.

Antero-inferior labral tear or soft tissue Bankart lesion is a frequent complication of anterior shoulder dislocation, commonly associated with Hill Sachs lesion. (Fig. 2) The dislocated humeral head can injure the glenoid labrum, sometimes resulting in an impaction fracture of the glenoid, referred to as bony Bankart. Impaction fracture of the posterior-superior humeral head is referred to as a Hill-Sachs lesion.

Other variants of this injury include Perthes, ALPSA, and GLAD.

Perthes type of injury involves chondral-labral detachment with periosteal stripping from the underlying glenoid without fragment migration. Another variant is anterior labroligamentous periosteal sleeve avulsion (ALPSA), where the chondrolabral and stripped periosteum migrate but remain attached to the glenoid. (Fig. 3) The GLAD injury involves labrum and articular cartilage damage.

MRI shoulder and MR arthrogram are the preferred modalities to diagnose these shoulder injuries. The defect/tear is a fluid-filled cleft on the fat-saturated T2 or proton density sequences.

### Elbow and forearm

The pediatric population is prone to fractures of the long bones, especially in contact sports such as American football. Common pediatric fractures include:

- Greenstick fracture (Fig. 7)
- Buckle fracture
- Spiral fracture (Fig. 5a)
- Volar plate avulsion fracture (Fig. 6)

Radiography is usually sufficient to diagnose these fractures; however, MRI may be valuable in cases of suspected ligament or cartilage injuries. On the other hand, CT is sometimes helpful in the surgical planning of complex fractures. Fracture healing is often characterized on radiographs by increasing periosteal reaction and callus formation. (Fig. 4) Serial radiographs are useful for assessing interval progression of healing, as well as for monitoring fracture alignment, stability, and proper osseous union over time.

### Lower extremity

One study suggested that extremities are the most common injury sites in pediatric football, with lower extremities accounting for 31–59% and upper extremities accounting for 21–34% of injuries. Pediatric injuries differ from adult injuries in that children have vulnerable sites such as ligament and tendons' attachment points, ossification centers and apophyses, and growth plates. In growing athletes, incomplete bone ossification leaves the growth cartilage at epiphyseal plates, articular surfaces, and apophyses softer and mechanically weaker than mature bone, making these regions more prone to stress and injury. [6]

Another study also highlighted the fact that lower extremity injuries were most prevalent in the two oldest groups (12–14 and 15–18 years), with rates of 33.0% and 33.6%, respectively, compared with the younger groups (< 8 and 8–11 years), which had rates of 26.5% and 31.8%. Similar findings were suggested by Mello et al. 2, who found a lower extremity trauma rate of 23.7% in 7 to 11-year-old players and a 27.3% rate in 12 to 17-year-olds and believed that the older and more experienced the youth athlete, the more refined the player's tackling technique will be. A decrease in "arm tackling" would be expected, and more focused forces toward proper tackling technique would lead to increased rates of injury from the waist down [3].

### Hip and thigh

Soft tissue injuries are common around the hip and thigh, including muscle hematoma, strain, muscle tear, Morel-Lavallée lesion, and soft tissue bruising. (Fig. 9).

Muscle hematomas demonstrate variable MRI appearances depending on their stage of evolution. Acute hematomas typically exhibit increased signal intensity on T2-weighted sequences, reflecting edema and the presence of fresh blood products. In the subacute phase, hematomas may appear as well-defined collections with peripheral rim enhancement on post-contrast T1-weighted images, corresponding to granulation tissue and inflammatory response. (Fig. 8).

Heterotopic ossification refers to the abnormal formation of mature bone within soft tissues, typically occurring in response to trauma or surgery. It most commonly develops in the periarticular or adjacent soft tissues near the site of an osseous injury. (Fig. 10).

Morel-Lavallee lesion is a distinct entity that involves the pre-fascial plane between the soft tissue and fascia. It results from the shearing force generated by mobile soft tissues and tough and nonmobile muscle fascia due to high-impact trauma, which tears the subdermal lymphatics and blood vessels.

MRI is the imaging modality of choice to diagnose Morel-Lavallee's lesion. The hemolymphatic collection can have different MR signal characteristics based on the composition. MRI can distinctly delineate the collection and its relationship with the underlying fascia. (Fig. 11).

### Knee

The knee is the most frequently injured joint in the lower extremity, followed by the ankle, shoulder, wrist, and hand [6]. Injuries include fractures and internal knee derangement involving anterior cruciate ligament (ACL) tear, meniscal and cartilage injury, and collateral ligament tear. Unlike adults, children more often sustain cruciate or collateral ligament injuries at their origin or insertion because the ligament–bone junction, weakened by incomplete ossification and growth cartilage, fails before the ligament itself, leading to avulsion injuries rather than mid-substance tears [6]. (Fig. 15).

The ACL connects the posterior femoral condyle to the anterior proximal tibia and prevents the anterior translation of the femur over the tibia. ACL injuries are commonly associated with medial compartment injuries involving the medial meniscus and medial collateral ligaments.

Other common injuries include isolated meniscal tears, patellar dislocation, osteochondral defects, and patellar cartilage defects. (Fig. 13, 14).

MRI knee is the modality of choice for diagnosing internal knee derangements. T2 fat-sat sequences will show increased signal intensity within the affected structure with or without distinct defects. (Fig. 16).

Intra-articular loose bodies can be identified as an avulsed bony fragment within the joint space in the case of an osteochondral defect. (Fig. 12).

### Ankle and foot

Ankle sprains account for nearly half of the pediatric sports related injuries with basketball (41.1%), football (9.3%), and soccer (7.9%) representing the most common mechanisms. Other types of injury include fractures, dislocation, and medial and lateral compartment ligament tear/sprain. [7] (Fig. 17).

Radiographs serve as initial screening tools for detecting fractures and dislocations. However, CT scans are often employed to identify occult fractures when findings on radiographs are inconclusive. MRI is particularly useful for evaluating ligament and cartilage injuries, as well as detecting CT- or radiograph-occult fractures, especially in patients with decreased bone mineralization. (Fig. 18).

### Orthopedic treatment approach for ACL reconstruction in pediatric patients

Surgical treatment for ACL reconstruction in an immature skeleton poses a significant risk of growth disturbances. The physis or growth plate is an essential bone segment responsible for the longitudinal growth of the long bones. It contains mesenchymal cells arranged in columns or layers, including the germinal or resting layer, proliferative zone, hypertrophic zone, and zone of provisional calcifications. Any trauma or surgery to the physis can damage the mesenchymal cells and could result in growth arrest.

Considering this, pediatric patients require different approaches to preserve the physis reserve.

The various types are as follows:

- Physeal-Sparing (Intra-articular)
- Partial Transphyseal (over the top)
- Partial Transphyseal
- Complete Transphyseal

### Physis-sparing ACL reconstruction

This technique involves autografts such as pes anserinus or iliotibial band to serve as an ACL, anchored on the femoral condyle proximally and anterior proximal tibia distally below the knee joint. The iliotibial autograft is cut in the middle, where the free end is passed over the top of the lateral femoral condyle. It enters the knee joint from the posterior surface of the knee and courses anteriorly. Finally, it exits beneath the anterior horn and is sutured at the proximal anterior tibia, sparing the physis.

### Complete trans-physis ACL reconstruction

This technique involves tibial and femoral tunnels with bone-tendon-bone configuration and the use of interference screws. Multiple reports suggest this technique improves knee function. [12] The majority of the studies included patients at least 14 years or older. The aim was to reach skeletal maturity before the surgery. Results showed improved knee function without evidence of growth disturbances or angular deformities.

Complications.

Literature reviews suggested a meager complication rate with the use of different surgical approaches for ACL reconstruction in pediatric patients [21]. Most complications result from surgical technique errors, such as hardware or suture placement at the level of physis. Some of the common complications are:

- Distal femoral valgus deformity
- Tibial recurvatum
- Limb overgrowth causing leg length discrepancy
- Re-rupture of the graft

Despite various treatment options, the treatment approach should be individualized based on the patient's age, activity level, and associated injury pattern. The general recommendations are to avoid tension in the graft, small tunnels perpendicular to the physis, fill the tunnels with soft tissue grafts, and prevent bone blocks or hardware at the level of physis [21].

## Data Availability

De-identified imaging data supporting the findings of this pictorial essay are openly available in the Zenodo repository at: 10.5281/zenodo.17625630.
